# Comprehensive Genomic Profile of Heterogeneous Long Follow-Up Triple-Negative Breast Cancer and Its Clinical Characteristics Shows DNA Repair Deficiency Has Better Prognostic

**DOI:** 10.3390/genes11111367

**Published:** 2020-11-19

**Authors:** Ernesto Rojas-Jiménez, Javier César Mejía-Gómez, Clara Díaz-Velásquez, Rosalía Quezada-Urban, Héctor Martínez Gregorio, Fernando Vallejo-Lecuona, Aldo de la Cruz-Montoya, Fany Iris Porras Reyes, Víctor Manuel Pérez-Sánchez, Héctor Aquiles Maldonado-Martínez, Maybelline Robles-Estrada, Enrique Bargalló-Rocha, Paula Cabrera-Galeana, Maritza Ramos-Ramírez, Yolanda Irasema Chirino, Luis Alonso Herrera, Luis Ignacio Terrazas, Javier Oliver, Cecilia Frecha, Sandra Perdomo, Felipe Vaca-Paniagua

**Affiliations:** 1 Laboratorio Nacional en Salud, Diagnóstico Molecular y Efecto Ambiental en Enfermedades Crónico-Degenerativas, Facultad de Estudios Superiores Iztacala, Tlalnepantla, Estado de México 54090, Mexico; erarroji@gmail.com (E.R.-J.); cdiazvelasquez@aol.com (C.D.-V.); rosalia.quezadaurban@petermac.org (R.Q.-U.); mag_hector@hotmail.com (H.M.G.); fernando.vallejo.leucon@gmail.com (F.V.-L.); literrazas@unam.mx (L.I.T.); 2 Unidad de Biomedicina, Facultad de Estudios Superiores Iztacala, UNAM, Tlalnepantla, Estado de México 54090, Mexico; audelacm@gmail.com (A.d.l.C.-M.); irasemachirino@gmail.com (Y.I.C.); 3 Division of Breast Cancer, Department of Medical Oncology, Mt. Sinai Hospital, University of Toronto, Toronto, ON M5G 1X5, Canada; javier.mejiagomez@mail.utoronto.ca; 4 Sir Peter MacCallum Department of Oncology, University of Melbourne, Melbourne, VIC 3000, Australia; 5 Cancer Research Division, Peter MacCallum Cancer Centre, Melbourne, VIC 3000, Australia; 6 Instituto Nacional de Cancerología, CDMX 14080, Mexico; fany.porras@gmail.com (F.I.P.R.); manuelps98@gmail.com (V.M.P.-S.); arzhaus@yahoo.com (H.A.M.-M.); ebargallo@yahoo.com (E.B.-R.); drapaulacabrera@gmail.com (P.C.-G.); maritzaramos1304@gmail.com (M.R.-R.); metil@hotmail.com (L.A.H.); 7 Hospital General de Pachuca SSA, Pachuca 42070, Mexico; roem1825@hotmail.com; 8 Instituto Nacional de Medicina Genómica, CDMX 14610, Mexico; 9 Unidad de Investigación Biomédica en Cáncer, Instituto de Investigaciones Biomédicas-Instituto Nacional de Cancerología, CDMX 14080, Mexico; 10 Medical Oncology Service, Hospitales Universitarios Regional y Virgen de la Victoria, Institute of Biomedical Research in Malaga, CIMES, University of Málaga, 29010 Málaga, Spain; javiom@gmail.com; 11 Unidad de Producción Celular del Hospital Regional Universitario de Málaga—IBIMA—Málaga, 29010 Málaga, Spain; frechacecilia@gmail.com; 12 Instituto de Nutrición, Genética y Metabolismo, Facultad de Medicina, Universidad El Bosque, Bogotá 110121, Colombia; perdomos@iarc.fr; 13 International Agency for Research on Cancer, World Health Organization, 69008 Lyon, France

**Keywords:** triple-negative breast cancer, whole exome sequencing, WES, treatment, somatic mutation, mutational signatures

## Abstract

Triple-negative breast cancer (TNBC) presents a marked diversity at the molecular level, which promotes a clinical heterogeneity that further complicates treatment. We performed a detailed whole exome sequencing profile of 29 Mexican patients with long follow-up TNBC to identify genomic alterations associated with overall survival (OS), disease-free survival (DFS), and pathologic complete response (PCR), with the aim to define their role as molecular predictive factors of treatment response and prognosis. We detected 31 driver genes with pathogenic mutations in *TP53* (53%), *BRCA1/2* (27%), *CDKN1B* (9%), *PIK3CA* (9%), and *PTEN* (9%), and 16 operative mutational signatures. Moreover, tumors with mutations in *BRCA1/2* showed a trend of sensitivity to platinum salts. We found an association between deficiency in DNA repair and surveillance genes and DFS. Across all analyzed tumors we consistently found a heterogeneous molecular complexity in terms of allelic composition and operative mutational processes, which hampered the definition of molecular traits with clinical utility. This work contributes to the elucidation of the global molecular alterations of TNBC by providing accurate genomic data that may help forthcoming studies to improve treatment and survival. This is the first study that integrates genomic alterations with a long follow-up of clinical variables in a Latin American population that is an underrepresented ethnicity in most of the genomic studies.

## 1. Introduction

Breast cancer (BC) is the most prevalent and lethal cancer in women worldwide [1]. It is clinically classified by the presence or absence of hormone receptors (estrogen receptor (ER) and progesterone receptor (PR)) and human epidermal growth factor receptor type 2 (HER2), which define the cancer subtype: Hormone-positive, HER2 positive, or triple-negative (TNBC). This classification is the gold standard procedure to decide the standard in each patient [2]. Targeted treatments such as trastuzumab, aromatase inhibitors, or selective hormonal receptor modulators have significantly improved the response and overall survival (OS) [3]. However, for TNBC, which comprises 10 to 15% of BC cases [4], the lack of expression of histological markers limits the capacity to treat the tumors, leaving chemotherapy as the only first line systemic treatment approved so far. Although the response is effective in 35% of patients [5], cases with residual disease have a higher recurrence and lower overall survival rates, reaching less than 50% in five years [6]. Furthermore, TNBC has a faster proliferation rate, higher histological grade, and a variable degree of tumor-infiltrating lymphocytes, which make these tumors more aggressive and the most associated by far with mortality among the three subtypes of BC [4].

Among Latin American patients, the frequency of TNBC has been shown to be higher than in other ethnic groups, reaching up to 14–20% [7,8,9,10]. Despite this higher prevalence in Latin American patients, there are very few genomic studies made on these population groups, most of them restricted to Latina women in the United States US [11].

The molecular complexity of TNBC is characterized by a marked molecular heterogeneity at expression and (epi) genomic levels [12,13,14]. Different omic approaches have attempted to define distinct subclasses of TNBC [12,13,15,16]; however, at the moment none has proven a practical clinical utility through independent replicative studies.

Nevertheless, multiomic studies have identified molecular properties of TNBC that can be exploited in therapy. One recurrent found alteration is DNA repair impairment, which suggests that combinations of alkylating platinum chemotherapy and targeted agents like poly(ADP-Ribose) Polymerase (PARP) inhibitors may offer a better prognosis [17,18,19,20]. Although some clinical trials have reported encouraging results, these treatments have not yet been incorporated into the clinical guidelines, due to the lack of sufficient evidence [2].

In this work, we characterized by exome sequencing (WES) the genomic alterations of 29 Mexican patients diagnosed with TNBC and associated their genomic profiles with various clinical characteristics in order to evaluate their role as predictive biomarkers of treatment response.

## 2. Materials and Methods

### 2.1. Patient Eligibility Criteria

A total of 97 patients diagnosed with TNBC and treated at the National Cancer Institute (INCAN) of Mexico were selected for TNBC diagnosis by immunohistochemistry (IHC) based on the following criteria: Less than one percent of the staining score for the ER, PR, and HER2 receptors [21]. All patients were diagnosed and treated between April 2007 and April 2010, and presented locally invasive tumors with neoadjuvant treatment, surgery, and evaluation of pathological complete response, which was defined as no histological residual cancer cells in the breast and axillary lymph nodes (ypT0/isypN0) [22]. After DNA quality control, only 29 samples were amplifiable and suitable for WES analysis (see below). All samples analyzed were treatment naïve. The protocol was approved by the Research and Ethics in Research Committees (protocol #016/013/IBI CEI/1021/16) and conducted in accordance with the Declaration of Helsinki. Samples were anonymized and sent to the Laboratorio Nacional en Salud: Diagnóstico Molecular y Efecto Ambiental en Enfermedades Crónico-Degenerativas, Facultad de Estudios Superiores Iztacala, UNAM.

### 2.2. Histological Assessment

All histopathological studies were performed by pathologists of the National Institute of Cancer (INCAN) Pathology Department. Receptor status for ER, PR, and HER2 was evaluated by IHC using standard ASCO/CAP guidelines [23], and fluorescent in situ hybridization for HER2 was carried out when appropriate. Tumor-infiltrating lymphocytes (TILs) status was assessed for each patient by H&E staining in the stromal, epithelial, and peritumoral compartments. Stromal infiltration was considered and classified as high or low with a ≥30% threshold. The pathologic complete response (PCR) was evaluated in accordance with the Residual Cancer Burden Index from MD Anderson.

### 2.3. Sample Preparation and DNA Extraction

Ninety-seven paraffin block primary biopsies, one from each case, were obtained from the pathology archive and about 50 mg of tissue was extracted from three 50-µm slices with a viable, non-necrotic tumor content of >90%. The sample was dewaxed with xylol and ethanol baths and digested overnight with proteinase K. The crosslinking formation by formaldehyde was reversed through 90 ºC incubations for 1 h. DNA extraction was done with the DNeasy Blood and Tissue Kit (Qiagen, Hilden, Germany) following the manufacturer’s instructions. DNA concentration was quantified with the Qubit dsDNA HS Assay Kit (Invitrogen, Carlsbad, CA, USA) and integrity and purity of the material was verified by agarose gel electrophoresis and spectrophotometry, respectively. Sixty-eight samples did not meet the minimum requirements for WES analysis and 29 were included in the study.

### 2.4. Clinicopathological Analysis

Patient information was collected and generated from electronic records. The collected information included (1) demographic data such as age at diagnosis, comorbidities, body mass index (BMI), previous pregnancies and parity, history of oral contraceptive use, hereditary breast cancer antecedents, type of treatment; (2) clinical outcome: Overall survival (OS), disease-free survival (DFS), PCR; and (3) tumor characteristics: Histological type, grade, and lymph node involvement. We assessed the survival probability in the 29 patients from the first day since the diagnosis to the last day of follow-up or death. Three patients (F39_133, F2_5, F85_261) that lacked the follow-up information were censored in survival analysis. The longest follow-up was 11 years.

### 2.5. Library Preparation

The library prep was done with the SureSelect XT Target Enrichment System for Illumina Paired-End Multiplexed Sequencing Library (Agilent Technologies, Santa Clara, CA, USA). Exonic regions were captured using SureSelect All Exon V6 kit (Agilent Technologies, Santa Clara, CA, USA) following the manufacturer’s instructions. Exonic regions, covering 60 Mb of protein-coding bases, were captured through hybrid captures. Sequencing was performed on an Illumina HiSeq 2500 by 125-base paired-end at Novogen, Sacramento, California.

### 2.6. Bioinformatic Analysis

Sequencing data was aligned with the reference genome hg19 using bwa-mem [24]. The GATK tools were used for the rest of the preprocess [25]. Duplicates were marked and the bases were recalibrated to generate BAM files that were finally called using Mutect2. The binary files were annotated using wANNOVAR (http://wannovar.wglab.org/) [26]. To filter pathogenic mutations, we considered AF > 0.05 with at least two reads on each strand to avoid strand bias. The mutations classified as pathogenic by ClinVar were selected [27]. For the rest of alterations we applied the following criteria: (1) A frequency < 0.001 in gnomAD [28] database and the Exome Sequencing Project (ESP6500) https://evs.gs.washington.edu/EVS/ [29]; (2) a prediction of deleterious alleles in at least two of the following mutational predictors: SIFT, PolyPhen-2, and Mutational Taster [30,31,32]. All mutations that fulfilled these criteria were classified as variants of uncertain significance (VUS). Driver genes were determined by Vogelstein and IntOGen cancer driver gene lists [33,34]. Genes that participate in immunologic pathways were classified according to previous reports [35,36,37,38]. All filtered mutations were manually curated by inspection of the BAM files with the IGV software (Broad Institute) [39]. All mutations were interpreted with the Cancer Genome Interpreter (CGI) tool (https://www.cancergenomeinterpreter.org/home) [40] from BBGLab to select driver mutations. CGI was also used to identify specific mutations with targeted treatment availability.

### 2.7. Tumor Mutational Burden Analysis

Tumor mutational burden (TMB) was defined as the number of somatic, coding, base substitutions, and indel mutations per megabase of genome examined. Known germline variations reported in dbSNP 151 were dismissed. To calculate the TMB per megabase, the total number of counted mutations was divided by the size of the coding region of the targeted territory. We used 60 Mb as the estimated size of the exome.

### 2.8. Mutational Signature Analysis

Mutational signature analysis was performed by the identification of the mutation fraction in each 96 of the trinucleotide context with R (version 3.5.1 Feather spray) package deconstructSigs developed by Rosenthal [41]. For OncoPrint analyses, a frequency threshold of ≥0.21 was considered as positive for signature 3. To classify the tumors by mutational signature patterns, we conducted an unsupervised hierarchical clustering analysis calculating Euclidean distances and grouping with Ward’s method [42].

### 2.9. Pathway Enrichment Analysis

For pathway and network enrichment analysis we used the David Functional Annotation Tool 6.8 (david.ncifcrf.gov) [43] using driver genes in KEGG pathway maps. We eliminated uninformative pathways, including general cancer and non-cancer diseases (infectious, addiction, and parasitic pathways).

### 2.10. Statistical and Survival Analysis

The Kaplan–Meier (KM) method was used to calculate all survival curves between clinical outcomes and molecular variables. The log-rank test was used to make comparisons between variables in the KM curves. A Wilcoxon test was applied to measure the differences between TMB and menopause status, proportion of mutational signatures, and overall survival. Finally, a linear correlation was applied to assess the association between signature 3 proportion and clinical outcome (OS and DFS) and age. All statistics were calculated with the R (3.6.2) packages survminer (0.4.6) and pvclust (2.2-0).

### 2.11. Global Actionable Alterations

Triple-negative tumors classified by immunohistochemistry for hormone receptors (estrogen and progesterone) and FISH amplification for HER2 were selected from the breast cancer studies at cBioPortal [44]. Information from the Nik–Zainal study was taken from the Supplementary Tables specific to the triple-negative status [45]. Global survival, mutation frequency data from the The Cancer Genome Atlas (TCGA) [46], METABRIC [47], and Nik–Zainal [45] cohorts were downloaded, and the mutations were annotated in the CGI [40]. Treatments approved by FDA for actionable genes, in all types of cancer, were selected using the CGI and OncoKB databases [40,48].

## 3. Results

### 3.1. Patient and Tumor Characteristics

We generated a cohort of 29 patients with an average follow-up of four years (range 0.7–11.1), and an average age at diagnosis of 50.1 (range 29–68). Family history of breast cancer was found in 10.34% of the patients. More than 72% of the diagnoses were made in stages II and III. The clinical characteristics of the 29 patients are summarized in Table 1. The patients’ treatment was based on the conventional approach (anthracyclines and/or taxanes) and other chemotherapies were added later (Table S1). We identified two general treatment groups: (1) Conventional chemotherapy, 66% (CCT; 19/29) and (2) conventional chemotherapy plus platinum salt derivatives, 34% (10/29; Ch-PLA).

### 3.2. Overall Survival and Complete Pathological Response

**Figure 1:**
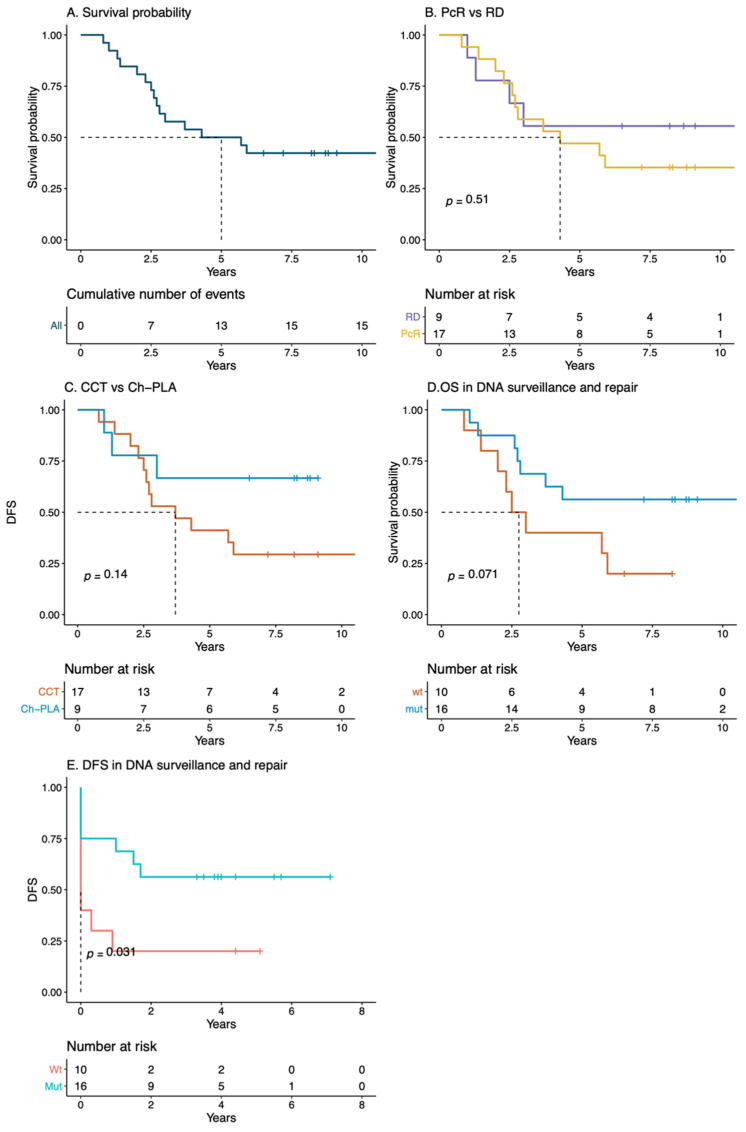
Overall survival (OS) probability (**A**) was assessed according to pathologic complete response (**B**) and therapeutic regime (**C**). The effect of deficiency in DNA repair and surveillance genes was evaluated for overall survival (**D**) and disease-free survival (**E**). PCR: Pathologic complete response; CCT: Conventional chemotherapy; Ch-PLA: Chemotherapy plus platinum salts.

The five-year OS was 50% (Figure 1A). There was a trend of better survival probability in patients with pathological complete response (Figure 1B). Between the groups of treatment, CCT, and Ch-PLA, there was no significant difference in survival probability (Figure 1C). Patients with mutations in DNA surveillance and repair genes (*TP53*, *BRCA1*, *BRCA2*, *ERCC6*, *FANCD2*, and *XPC*) showed a trend of better OS (Figure 1D). Finally, we found an association between deficiency in DNA repair and surveillance genes and DFS (Figure 1E).

### 3.3. Mutational Burden

**Figure 2:**
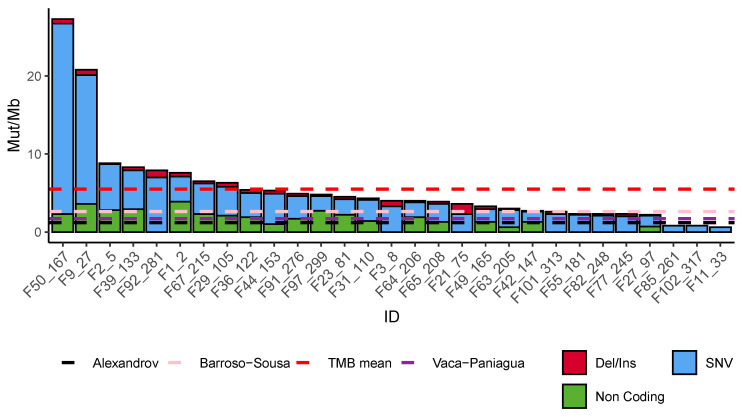
Tumor mutation burden (TMB) on each sample according to the type of variant. The type of mutation is indicated: Indels and deletions (red), single nucleotide variations (SNVs) (blue), and non-coding (green). The red dotted line is the mean TMB of our cohort and the black and purple dotted line is the TMB mean found by Alexandrov (2013), Vaca–Paniagua (2015), and Barroso–Sousa (2020) [49,50,51].

Overall, we identified a total of 2,690,172 mutations. All intronic, SNPs (>0.001), and low-quality sequences were discarded, leaving 10,040 mutations. The distribution of mutations among samples was heterogeneous with a high frequency of single nucleotide variations (SNVs) (70.3%), followed by non-coding mutations (splicing and ncRNA; 24.4%), insertions, and deletions (5.11%). The mutational burden also showed a heterogeneous distribution with a median of 5.52 mutations per Mb (interquartile range 2.5–6.2/Mb) (Figure 2). From all the mutations found, only 51 pathogenic alterations were detected on driver genes (see Materials and Methods). Menopausal status, PCR, treatment, OS, and age were not associated with the mutational burden (Figure S1A–F). The highest mutational burden was found in a tumor with a pathogenic mutation in *MSH6* (F50_167).

### 3.4. Somatic Mutational Landscape

**Figure 3:**
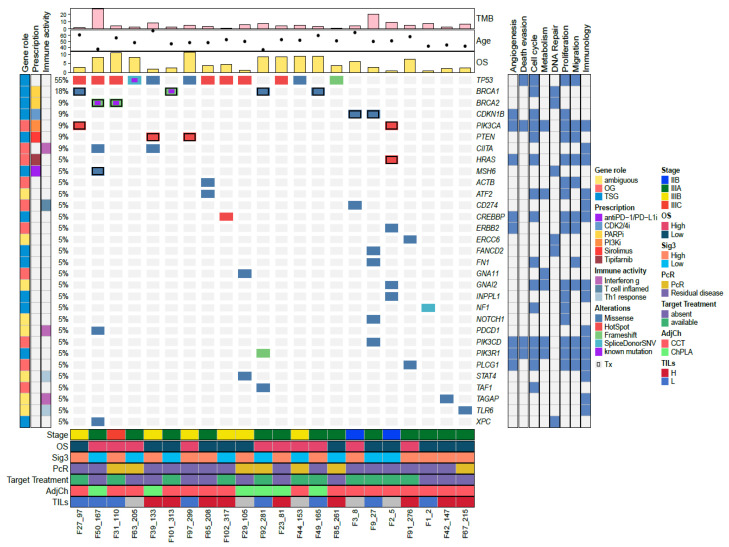
Allelic composition and molecular characteristics of the triple-negative breast cancer (TNBC) samples. Driver mutations are depicted according to the type of mutation (missense, hot spot, frameshift, splicing). Specific mutations already reported in other TNBC studies are shown with the label “known mutation”. Treatment availability for specific mutations is marked with a black frame. The functions of the driver gene were classified as oncogene (OG), tumor suppressor gene (TSG), or ambiguous, according to Vogelstein (2013) and IntOGen [33,34]. Prescription availability (clinical trials or FDA approved) for the specific mutations detected is represented according to molecular pathway targeted; *TP53* treatment is under early clinical trials and not currently approved by FDA; genes that participate in immunology pathways were classified as INF-γ (interferon g), T cell inflammation activity (T cell inflamed), and Th1 response (Th1_response). The lower panel shows the annotations of clinical characteristics (stage, OS, PCR), potential targeted treatment availability (target treatment), the use of platinum salts in the neoadjuvant setting (NeoCh), and the presence of tumor-infiltrating lymphocytes (TILs). The pathways in which each gene participates are shown at the right-side panel (see Materials and Methods). The top plots illustrated OS, age at the diagnosis, and TMB.

Overall, we detected 31 driver genes with pathogenic mutations, from which 41.9% (8/31) were classified as tumor suppressors, 32.3% (10/31) as oncogenes, and 25.8% (8/31) as having an ambiguous function, according to the CGI [40] (Table S2). In seven samples we did not detect any driving mutation. Moreover, the most frequent mutations were found in *TP53* (41.4%, 12/29), followed by *BRCA1* (13.8%, 4/29) and *BRCA2* (6.9%, 2/29) (Figure 3). Of importance, eleven out of the 29 patients (37.9%) had a specific mutation associated with an available targeted treatment (Figure S2A). These driver mutations with possible actionable treatments were found in *BRCA1, BRCA2, CDKN1B, PIK3CA, PTEN*, and *HRAS* (Figure S2B). The potential therapeutic drugs already approved by FDA are PARP inhibitors (PARPi) for *BRCA1/2* mutations, tipifarnib (farnesyltransferase inhibitor) for *HRAS*, and inhibitors of *PIK3CA* hotspot mutations E545K and H1047R and sirolimus (MTOR inhibitor) for *PTEN* mutations. In addition, there were two patients with mutations in *CDKN1B* which are potential markers for CDK2/4 inhibitors (Table S3). Importantly, patients with *BRCA1*/*2* mutations showed a trend for improved OS; however, the low frequency of patients did not allow for a statistical significance to be reached (Figure S3).

### 3.5. Tumor Infiltrating Lymphocytes

Infiltrating lymphocytes assessed by histopathology showed 13 high and 10 low infiltration tumors. Six samples could not be evaluated (Figure 3, lower panel). The TILs were analyzed for association with the clinical and molecular variables, but there were no significant associations.

### 3.6. Mutational Signatures

**Figure 4:**
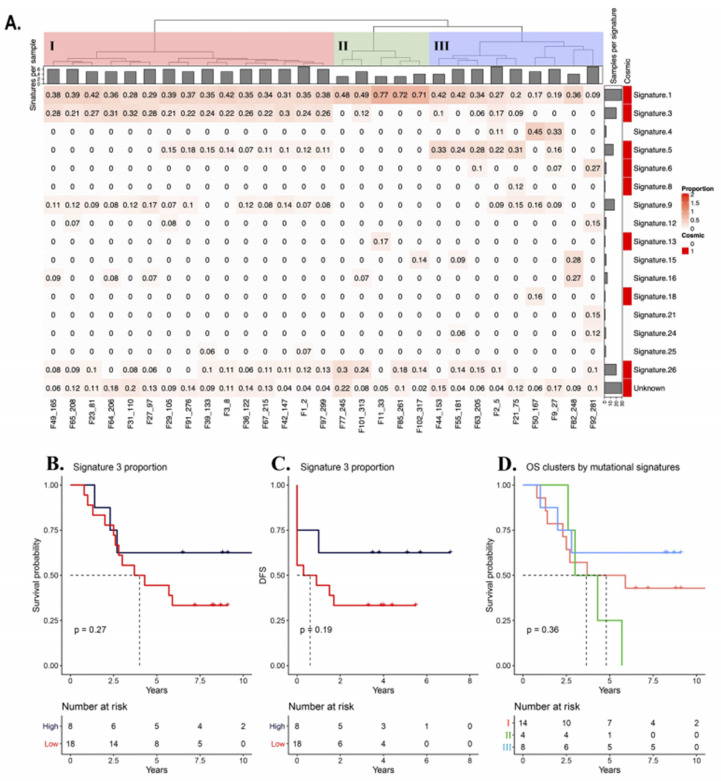
Tumor genomic distribution according to mutational signature profile and overall survival probability of each group. (**A**) Heatmap of the mutational signatures clustered with the Ward’s method (see Materials and Methods section). Three different mutational signature groups were identified and defined as I, II and III. (**B**) Overall survival probability of according to signature 3 proportion (high versus low). (**C**) Disease-free survival probability according to signature 3 proportion (high versus low). (**D**) Overall survival probability of the three mutational signature groups.

We identified the operative mutagenic processes and quantified their proportion on the tumor exome by mutational signature analysis. Sixteen out of the 30 mutational signatures reported by Alexandrov (2013) were detected [49]. On one hand, the most represented signatures were: 1, 3, 5, 9, and 26, at least present in 15 tumors (Figure 4A). One of the molecular attributes of TNBC is a higher frequency of homologous recombination (HR) deficiency, which in turn has been associated with signature 3. We found an association between the high proportion of signature 3 (>0.21), OS, and DFS, but without statistical significance (Figure 4B,C).

We clustered all the samples to define groups based on their mutational signature composition. Three large groups (I, II and III) were detected (Figure 4A). Group I was defined by the highest proportion of signature 3, including all samples having a frequency above 0.2. Group II had samples with the highest proportion of signature 1, practically lacking signature 3 and enriched in signature 26. Group III was the most heterogeneous of all, composed of two subgroups, the first one enriched for signature 5 and the second one with the most heterogeneous signature distribution. To search for an association between the groups with clinical outcome, we calculated the OS probability according to each group. We found that group II had the lowest OS, but the results were not statistically significant (*p*-value = 0.36; Figure 4D).

Furthermore, we evaluated the association of the OS status with the proportion of each mutational signature. Given that 50% of the patients survived after 5 years, we used this number as the threshold to define two groups: High OS (>5 years) and low OS (<5 years). After elimination of the censored patients, the groups were 44.8% (13/29) with high OS and 55.2% (16/29) with low OS. Signature 3 did not show association with PCR (Figure S4). Signatures 18, 21, and 24 were detected exclusively in patients with longer OS, whereas signature 8 and 25 were present only in patients with shorter OS. However, we found no statistical association between these signatures and the OS status (Figure S5).

Moreover, the ubiquitous signatures 1 and 5 were commonly identified in the entire cohort and in 15 samples, respectively. Signature 1 and signature 5, which are both linked to aging, contributed with 9–77% and 7–33% of the total number of mutations, respectively. However, we did not observe a linear association between age and the proportion of the signature 1 and 5 (Pearson *R* = 0.28 and 0.22). Figure S6A,B).

We then evaluated the association of TMB with proportions of the five repair mutational signatures detected (signature 3 for HR deficit and signatures 6, 15, 21, and 26 of mismatch repair (MMR) impairment) (Figure S6C–G). However, we did not find any significant association (signature 3 R = 0.17, signature 6 R = 0.24, signature 15 R = 0.4, signature 21 R = 0.23, signature 26 R = −0.31).

In addition, we noticed that roughly half of the samples presented a proportion ≥0.2 for signature 3 (all group I, *n* = 15/29), and the other half had ≤ 0.2 of this signature (groups II and III, *n* = 14/29). We evaluated the possible association of OS and DFS with higher (≥0.2) and lower (≤0.2) proportions of signature 3 (Figure S7A,B). We found no association between signature 3 and these clinical variables.

Finally, a higher proportion of signature 5 (≥0.14) was characteristic of group III, and a higher proportion of signature 26 (≥0.16) was distinctive of group II. We used the threshold values of these signatures to categorize the samples and to evaluate their association with OS and DFS. Overall, there was no significant difference between higher proportions in signatures 3, 5, and 26 and OS, DFS, or PCR (Figure S7C–I). With these analyses we conclude that there is no significant association of mutational signatures with clinical outcome. Likewise, the three mutational signature groups were not associated with age at diagnosis, OS, DFS, and TMB (Figure S8A–D).

### 3.7. Molecular Pathways

**Figure 5:**
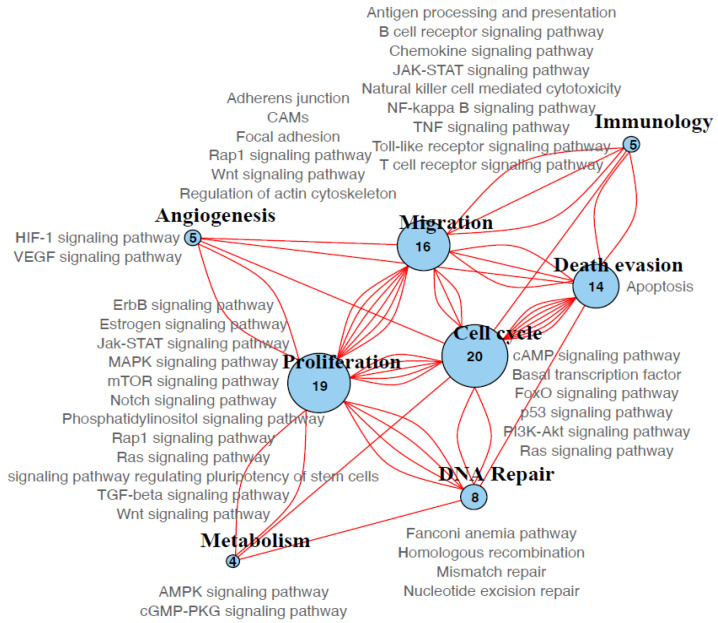
Global biological processes enriched in the tumors. Eight large processes of enriched pathways were detected. The size of the circle equals the number of genes associated with each process across all samples. Lines indicate that the same gene participates in the linked processes. The pathways grouped in each process are listed. The number of genes and pathways that participate in each process is indicated.

After performing a functional pathway enrichment analysis, we identified 158 different KEGG pathways from which 41 remained after filtering non-informative pathways (see Materials and Methods). Then, we grouped and classified eight different pathways or processes: Proliferation, cell cycle, migration, immunology, angiogenesis, DNA repair, metabolism, and death evasion (Figure 5). The most prevalent process was proliferation which was found in every sample analyzed. Cell cycle pathway was the second most frequent, followed by migration and death evasion (Figure 5).

### 3.8. TNBC Potential Therapy Perspective

**Figure 6:**
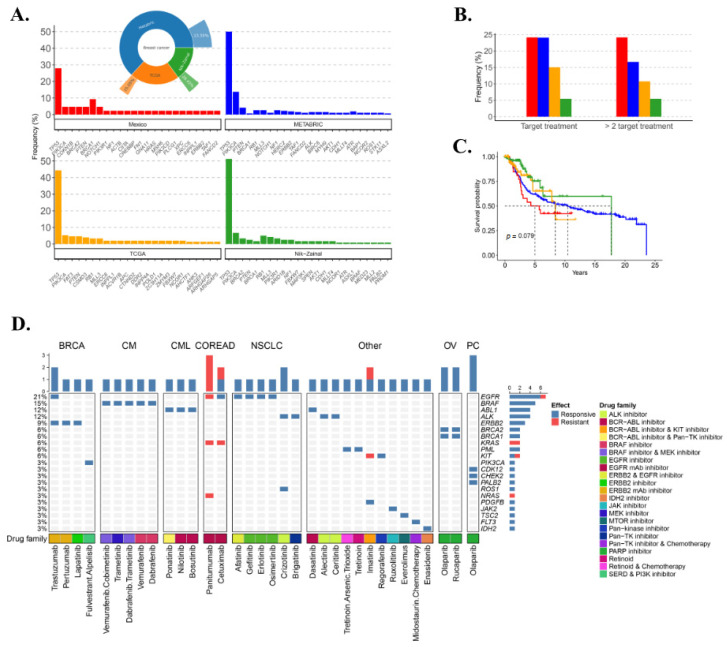
Distribution of target treatments in the Mexican cohort, METABRIC, TCGA, and Nik–Zainal TNBC. (**A**) Frequency of the top 24 genes mutated for every cohort. Inset: Frequency of TNBC cases in METABRIC, TCGA, and Nik–Zainal cohorts. (**B**) Overall survival comparison between the four cohorts in locally advanced tumors. (**C**) Frequency of patients with at least one potential target treatment and those with two or more treatments. (**D**) Composition of markers for response or resistance for all FDA-approved drugs in the four cohorts and the type of tumors for which they have been approved. BRCA: Breast cancer, CM: Cutaneous melanoma, CML: Chronic myeloid leukemia, COREAD: Colorectal adenocarcinoma, NSCLC: Non-small cell lung, OV: Ovary, PC: Prostate cancer, other tumors (AML: Acute myeloid leukemia, APML: Acute promyelocytic leukemia, GIST: Gastrointestinal stromal, LUAD: Lung adenocarcinoma, MY: Myelofibrosis, ST: Stomach, GEJA: Gastro-esophageal junction adenocarcinoma, HES: Hypereosinophilic advance syndrome, ALL: Acute lymphoblastic leukemia, MDS: Myelodysplastic syndrome, SM: Systemic mastocytosis, MDPS: Myelodysplastic proliferative syndrome, RA: Renal angiomyolipoma, DFS: Dermatofibrosarcoma, ECL: Eosinophilic chronic leukemia, LUAD: Lung adenocarcinoma, GCA: Giant cell astrocytoma). The target pathway is depicted.

To evaluate the distribution of pathogenic variants in larger cohorts, we obtained data from METABRIC, TCGA, and Nik–Zainal databases (see Material and Methods). We selected 320 out of 2369 (13.5%) TN tumors from TCGA, 123 out of 817 (15.05%) from METABRIC, and 148 out of 560 (26.43%) from the Nik–Zainal study. We identified the most frequently mutated driver genes and found coincidences in genes such as *TP53*, *PIK3CA*, and *PTEN* among all studies (Figure 6A). We evaluated the OS in locally advanced tumors (IIa-IIIc). A Kaplan–Meier curve was made with a statistical comparison by log-rank test between the three cohorts, and no significant difference was observed (Figure 6B). In the three studies, 21.13% of the tumors analyzed had at least one target treatment available, approved by the FDA, and 15.49% had more than two (Figure 6C). Finally, we analyzed the distribution and composition of actionable mutations of the previously filtered FDA treatments (Table S4). We found 31 single targeted treatments or combinations for 23 types of cancer. Importantly, the PARP inhibitors olaparib and rucaparib, which are approved for ovarian cancer and prostate cancer, have already been tested on TN tumors (Figure 6D) [17,18,19,20]. We looked for clinical trials (terminated or completed) on BC for approved treatments in other cancers that target the driver mutations detected in the three cohorts, and found 103 trials and 15 drugs tested (Trametinib, Vemurafenib, Crizotinib, Cetuximab, Panitumumab, Afatinib, Gefitinib, Erlotinib, Tretinoin, Imatinib, Sunitinib, Ruxolitinib, Dasatinib, Bosutinib, Everolimus) (Table S5). Everolimus, Sunitinib, and Gefitinib are the most tested drugs, with 62, 28, and 23 studies terminated or completed. These findings demonstrate that TN tumors present potentially actionable alterations in at least 21% of the cases, and are distributed in 15 genes with 15 potential associated drugs.

## 4. Discussion

TNBC is a highly heterogeneous disease, clinically, molecularly, and in terms of prognosis. This heterogeneity is the main challenge to search for a successful treatment and for OS extension. In this work, we characterized the genomic alterations of a set of Latin American TNBC cases and compared these changes with relevant clinical variables. We analyzed 29 treatment-free tumor samples from primary diagnosis from each patient. Clinical data agreed with those previously reported in epidemiological studies: Younger age of diagnosis (mean 50.1), ranging between 29 and 68; family history of breast cancer in 10% [4,9]. Globally, these patients had a poor prognosis, and a five-year overall survival of 50%, which is lower as compared to results reported by Reynoso in 2019 in 4300 patients treated in Mexico’s INCAN, which showed 82% for all BC cases and 75% for the TNBC subtype [52]. Although Reynoso’s study comes closer to the survival probability found in other studies reporting survival probabilities above 75% [53,54,55], we could explain our discrepancies based on differences in the study set up. For instance, the difference in OS probability in our cohort could be caused by the fact that we selected only locally advanced tumors. Indeed, more than 82% of them were in stages IIIa and IIIb, in contrast to the study of Reynoso, which had a distribution of 36.6% patients in stage II and 36.2% in stage III. However, a report from the Surveillance, Epidemiology, and End Results Program (SEER) database showed that the OS of stage III TNBC was <60% [3], in agreement with our results.

The neoadjuvant treatment has been a cornerstone for OS extension in BC. For TNBC there is no approved targeted alternative for neoadjuvant therapy [2] and different therapeutic approaches are currently being tested. From 286 TNBC ongoing clinical trials, only 76 have been completed with available results (clinicaltrials.gov). Of these trials, platinum-based treatments are one of the most common drugs used as intervention in the neoadjuvant setting. While there is evidence supporting the use of platinum derivatives in TNBC, the National Comprehensive Cancer Network (NCCN) has not yet considered it safe for the use in the neoadjuvant milieu, because of a lack of significant clinical evidence [2]. In our study, we classified our patients into two groups, depending on whether they received platinum therapy in addition to their conventional therapy. We took OS and PCR as a surrogate biomarker for prognosis. Patients with PCR had better survival as compared with patients with residual disease, but the difference was not statistically significant. Regarding treatment, although the clinical outcome showed a trend to higher PCR for patients treated with platinum, there was no significant difference between the two groups. Other studies have observed the same clinical response [5,56,57], and some have reported significant differences [58,59,60,61].

The mutational architecture of the analyzed tumors showed important differences. TMB ranged from over 30 mutations per Mb to only one, with a mean of 5 mut/Mb, and a predominance of single nucleotide mutations. This value is higher than those reported by Alexandrov in 2013, Vaca–Paniagua in 2015, and Barroso–Sousa in 2020 (1.2 mut/Mb, 1.7 mut/Mb, and 2.65 mut/Mb, respectively) [49,50,51], although in this last study, TNBC showed a higher mean TMB than other BC subtypes [51]. Furthermore, we identified driver mutations in 31 genes from which 13 were tumor suppressors, 8 were oncogenes, and 8 were genes with an ambiguous role in tumorigenesis. The most frequent driver genes found mutated were: *TP53, BRCA1, BRCA2, PIK3CA*, and *PTEN*. This result is consistent with large genomic studies on BC, including TCGA [62], METABRIC [47], and MSK-IMPACT [63], and with other TNBC from us and others [14,50,64,65,66]. In addition, mutations were found in *ERCC6, GNA11*, and *XPC* that have not been identified in previous reports of TNBC tumors. Each one of these mutations was found separately in different patients. Regarding the roles of these genes, *ERCC6* codes for a DNA-binding protein that participates in transcription-coupled nucleotide excision repair and nucleotide excision repair of DNA. *GNA11* codes for guanine nucleotide-binding transducer of transmembrane signaling that is highly mutated in uveal melanoma [67]. Mutations in the nucleotide excision repair gene *XPC* are causal of the autosomal recessive disease Xeroderma pigmentosum [68]. The presence of these alterations reflects the highly heterogeneous mutational nature of TNBC and it is possible that some of these alterations precede neoadjuvant treatment as has recently been demonstrated [69]

Pathogenic mutations that are markers for targeted treatment were found in 38% (11/29) of the patients. These alterations include pathogenic mutations in *BRCA1/2,* which are used for PARP inhibitors [70,71,72], and in *PIK3CA* which is a marker for PI3K pathway inhibitors. The clinical utility of these mutations is currently being tested in early trials [73,74,75]. Other potentially targetable mutations were found in *TP53* [76], *HRAS* [77,78], *PTEN* [79], and *CDKN1B* [80], which are markers used in clinical trials and the preclinical stage. The prevalence of these mutations may broaden the alternatives to targeted therapies, although carefully designed clinical studies are still needed to prove their clinical value.

We evaluated if *BRCA1/2* was associated with platinum sensitivity. No association was found in PCR, DFS, and OS, although overall patients treated with platinum had better OS. Other genomic studies on TNBC have reported that *BRCA1/2*-deficient tumors have a greater benefit from platinum-based treatments [20,81]. Likewise, phenocopy of *BRCA1/2* alterations have been shown to impair HR. This state has been termed BRCAness [82], and has been shown to be associated with platinum sensitivity. In this study we found no significant association between PCR, OS, and DFS with signature 3, which was used as a surrogate of BRCAness. Despite there being no association between mutations and signature 3 with clinical response and prognosis, the high frequency of patients showing these alterations (31% (9/29)) suggests that a combination of platinum chemotherapy and PARP inhibitors may be a potential treatment in the neoadjuvant setting for TNBC. Of importance, there is a clinical study in course to test safety and efficacy of this combination NCT03150576 (clinicaltrials.gov).

Moreover, we found 16 operative signatures in the tumors analyzed, eight of them had been previously reported on BC (signatures 1, 3, 5, 6, 8, 13, 18, 26) and the other eight were not yet reported (signatures 4, 9, 12, 15, 16, 21, 24, and 25) [45,49]. The etiology of the found mutational signatures was related to: Mutational process by spontaneous deamination (signature 1), APOBEC activity (signature 13), DNA repair mechanism impairment (signature 3, 6, 15, 21, and 26), polymerase eta failure (signature 9), exposure to exogenous mutagens (tobacco and aflatoxins signatures 4 and 24, respectively). Six of the signatures (signatures 5, 8, 12, 16, 18, 25) have no known etiology. Of note, signature 26 is attributed to deficiency in the DNA MMR mechanism that is a state which results in high mutational load [45]. Interestingly, the sample with the highest mutational burden, 167, had alterations in *MSH6* and *XPC*, but lacked signatures attributed to MMR.

Pathogenic variants in genes that have a role in DNA repair and surveillance, as well as high proportion of mutational signature 3, associated with impairment in HR are a hallmark of TNBC biology [45,82,83]. We observed a high frequency (65%, 19/29) of pathogenic variants in *TP53, BRCA1, BRCA2, MSH6, ERCC6*, and *XPC*, and signature 3 in these samples. These alterations showed a clear trend for better clinical outcome regarding OS and DFS (Figure 1D,E, Figure 6B,C, and Figure S3). An increase in chemotherapy sensibility, and higher OS and DFS were associated with these molecular changes in several reports [83]. While our analyses did not show statistical differences, our findings are consistent with two similar larger multiomic studies of TNBC; one in 254 samples from the Sweden Cancerome Analysis Network—Breast (SCAN-B) [84] and the second in 456 samples from the Fudan University of Shanghai Cancer Center (FUSCC) [85]. Along with the detection of similar mutated genes, they also performed the HRDetect algorithm [84], for the identification of DNA HR deficiency. As found in our study, both reports showed a better prognosis when the HR score was classified as high.

Furthermore, we tried to identify the immunological profile of TNBC patients. Interestingly, other studies have reported that the TN subtype showed the highest lymphocyte infiltration (TILs) among breast cancer, due to the heterogeneous microenvironment. An active immune response involving high levels of TILs has been associated with a better prognosis and a better response to treatment [86]. Here we identified samples with high and low TILs from the pathology report and identified six genes associated with immunological responses according to Hutchinson [87]: *STAT4* and *TLR6* with Th1 response, *CIITA, TAGAP, PDCD1*, and *TLR6* with an interferon-γ response and *CD247* with a T cell-inflamed response. Despite the observed trend, neither TILs nor the alteration of immunological genes was associated with the clinical outcome.

An important limitation of this study was the small number of patients included, which impacted the statistical associations between the molecular and clinical variables. Another limitation was the lack of paired normal tissue, which was unavailable or had low DNA quality in the majority of the cases. This constraint may have impeded the identification of germline pathogenic variants, may cause the potential wrong imputation of common variants as somatic mutations, and may also result in the incorrect attribution of pathogenic mutations produced by clonal hematopoiesis as tumor mutations. To filter out non-pathogenic natural variation, we used the gnomAD and ESP6500 [28,29] repositories in our filtering algorithm. These databases collectively contain genetic information of 78,205 individuals, including more than 17,720 Latinos, most of them of Mexican origin. Furthermore, the gnomAD repository is not overrepresented for pathogenic variants, which supports its use to estimate normal variation [88]. This strategy is widely used in tumor-only studies similar to ours to reduce the effect of a lack of paired-controls [89,90]. In addition, this design did not allow us to perform methylation analysis, which could have provided a more complete molecular panorama. Promoter hypermethylation is a well-known mechanism of transcriptional repression of tumor suppressor genes in breast cancer, including *BRCA1, APC, CDH1, FANCF,* among others, and may provide a complementary pathway of molecular carcinogenesis, independent of mutations [91,92,93,94]. Furthermore, *BRCA1* promoter hypermethylation has been shown to be a common early event in the development of TNBC, and epigenetic silencing of *RAD51C,* which is also strongly associated with signature 3 in basal-like breast cancer [95,96]. We will address these constraints in future studies.

The large TNBC intertumoral heterogeneity at the genetic level shown here deserves an additional effort, since multiple markers for directed treatment can be used. In this study, we found 29 targeted treatments that can be exploited according to the vulnerabilities of each tumor, of which 15 have been evaluated in clinical trials for BC. Some drugs such as Everolimus [83,97,98,99,100,101] and Gefitinib [102] have already begun to be tested with encouraging results for TNBC in combination with other drugs. The results of these studies will provide valuable information on new therapeutic approaches for this disease. Another 13 drugs have already completed clinical trials (See Table S5). Larger clinical studies are needed to recognize and address the various genetic vulnerabilities of TNBC. Different TNBC studies have tried to identify genomic patterns or molecular alterations, but at the moment there is no single molecular marker of clinical utility [13,14,16,45,103,104]. This is a direct result of the notorious molecular complexity of this disease. Our findings provide further detailed evidence on the unique molecular and genomic heterogeneity of TNBC in a population underrepresented in genomic studies and pinpoint DNA repair deficiency as a potential marker of better prognosis. Our results suggest that molecular attributes beyond mutational status need to be considered to better identify the exploitable underlying vulnerabilities of TNBC. This compendium of molecular vulnerabilities may be dynamic and change over time during the tumor evolution. Large clinical studies are still necessary to define composite molecular markers of clinical value.

## 5. Conclusions

We performed a comprehensive genomic profiling of TNBC through the integration of molecular analyzes, including mutations and the composition and proportion of operative mutational signatures with relevant clinical variables, which allowed us to better characterize the molecular complexity of this disease. These tumors showed a substantial molecular heterogeneity and a better prognosis when DNA repair deficiency was present. Our findings provide detailed information on the specific and global alterations of TNBC in a currently understudied population. Moreover, this study contributes to further elucidate the global molecular alterations of TNBC, by providing precise genomic data that may help forthcoming studies to improve treatment, survival, and quality of life of these patients.

## Figures and Tables

**Table 1:** Clinical characteristics of the patients (*n* = 29).

Age at the Diagnosis		Hereditary Breast Cancer	
Mean	50.1 (± 8.72)	Yes	3 (10.34%)
Range	29–68	No	26 (89.66%)
Menopausal status		Tumor stage at diagnosis	
Premenopausal	10 (34%)	IIB	5 (17.24%)
Postmenopausal	16 (55%)	IIIA	14 (48.28%)
Co-morbidities		IIIB	7 (24.24%)
Diabetes mellitus	7 (24%)	IIIC	3 (10.34%)
Systemic hypertension	7 (24%)	Pathological complete response	
BMC *		PCR	10 (34.48%)
Mean	27.94 (± 5.10)	Residual Disease	19 (65.52%)
Previous pregnancy		Neoadjuvant chemotherapy with platinum salts	
Yes	24 (82.76%)	Yes	10 (34.48%)
No	5 (17.24%)	No	19 (65.51%)
Oral contraceptives		DFS * (years)	
Yes	7 (24.24%)	Mean	2 (± 2.24)
No	22 (75.86%)	Range	0–7.1
OS * (years)			
Mean	5 (± 2.24)		
Range	0.7–11.1		

* BMI = Body mass index, OS = Overall survival, DFS = Disease-free survival.

## Supplementary Materials

The following are available online at https://www.mdpi.com/2073-4425/11/11/1367/s1, Table S1: Chemotherapy treatment by patient. Table S2: Driver mutations by patient. Table S3: Potential targeted treatments. Table S4: Potential targeted treatments approved by FDA in our cohort, METABRIC and TCGA. Table S5: Clinical trials for FDA-approved drugs on BC tumors. Figure S1: Association between clinical outcome and TMB, Figure S2: Targeted treatments, Figure S3: Overall survival as a function of BRCA1/2status, Figure S4: Association between clinical outcome and signature 3, Figure S5: Comparison between the OS and the signature proportion, Figure S6: Lineal correlation between signature proportion and clinical and molecular variables, Figure S7: Association between threshold of the signature proportion and the clinical outcome, Figure S8 differences between groups according to clinical and molecular outcome.
