# Supplementary material for: Comprehensive Genomic Profile of Heterogeneous Long Follow-Up Triple-Negative Breast Cancer and Its Clinical Characteristics Shows DNA Repair Deficiency Has Better Prognostic
Source: Genes (Basel). 2020 Nov 19;11(11):1367. doi: 10.3390/genes11111367 (PMC7699204; doi:10.3390/genes11111367)
Supplement: Supplementary file 1 [file genes-11-01367-s001.pdf]

Supplementary material

TABLES

Supplementary Table 1. Chemotherapy treatment by patient.

ID	Chemotherapy status	Neoadjuvant chemotherapy	Adjuvant chemotherapy	Palliative treatment
F1_2	CCT	FAC+Paclitaxel+RT	NA	NA
F101_313	CCT	ADR+Paclitaxel+RT	Capecitabine	NA
F102_317	CCT	FAC+Paclitaxel+RT	Capecitabine	NA
F11_33	CCT	NA	CDDP+Paclitaxel+ GMZ+CBP	NA
F2_5	CCT	ADR+FOS+RT+Tamoxifen	Paclitaxel+CDDP+CBP	Docetaxel/GMZ
F21_75	CCT	FAC+Paclitaxel+RT	CBP	Capecitabine
F23_81	ChPLA	ADR+CDDP+Paclitaxel	RT+Capecitabine	NA
F27_97	CCT	FAC+Paclitaxel+RT	NA	Capecitabine
F29_105	ChPLA	FAC+CDDP+Paclitaxel+GMZ+RT	NA	CBP+Docetaxel+ Capecitabine
F3_8	CCT	FAC+Paclitaxel+Capecitabine	Capecitabine+CBP+ Navelbine	NA
F31_110	CCT	FAC+Paclitaxel	RT	NA
F36_122	ChPLA	FAC+CDDP+Paclitaxel	RT	NA
F39_133	ChPLA	CDDP+Paclitaxel	RT+CDDP+GMZ+Capecitabine+CBP	NA
F42_147	CCT	FAC+Paclitaxel	CDDP+Navelbine	GMZ+CBP
F44_153	CCT	FAC+Paclitaxel	RT+CDDP+GMZ	NA
F49_165	ChPLA	CDDP+ADR+Paclitaxel	RT+Capecitabine	NA
F50_167	ChPLA	CDDP+DOXO+Paclitaxel	RT+Capecitabine	NA
F55_181	ChPLA	FAC+Paclitaxel+CDDP	NA	NA
F63_205	CCT	FAC+Paclitaxel	NA	NA
F64_206	CCT	FAC+Paclitaxel+RT	Navelbine	CDDP+GMZ
F65_208	CCT	FAC+Taxol	Capecitabine	CBP+Docetaxel
F67_215	CCT	FAC+Paclitaxel+GMZ+RT	NA	Capecitabine+Zometa+Xeloda+Navelbine
F77_245	ChPLA	FAC+Paclitaxel+CDDP+GMZ+RT	Capacitabine	NA

F82_248	ChPLA	FAC+Paclitaxel+CDDP	RT	NA
F85_261	CCT	FAC+Paclitaxel	RT	Capecitabine+CBP+GMZ
F9_27	CCT	FAC+Paclitaxel+GMZ+RT	Capecitabine+Navelbine	NA
F91_276	CCT	FAC+Paclitaxel	RT	NA
F92_281	ChPLA	CDDP+ADR+Paclitaxel+RT	NA	NA
F97_299	CCT	FAC+Paclitaxel+RT	Capecitabine	NA

Abreviations: CCT: conventional chemotherapy, ChPLA: conventional chemotherapy plus platinum salt derivates plus platinum salts, FAC: fluorouracil/adramycin/citoxan, RT: radiotherapy, ADR: adramycin, GMZ: gemzar, CBP: carboplatin, FOS: cyclophosphamide, CDDP: cisplatin, DOXO: doxorubicin, NA: not available.

Supplementary table 2. Driver mutations by patient.

ID	gene	Protein	HotSpot	gene_role
F1_2	*NF1*	splicing	No	Tumor suppressor gene
F101_313	*BRCA1*	G1077Afs*8	No	Tumor suppressor gene
F102_317	*CREBBP*	R1446C	Yes	Tumor suppressor gene
F102_317	*TP53*	R213*	No	Tumor suppressor gene
F2_5	*ERBB2*	R849Q	No	Oncogene
F2_5	*HRAS*	G12D	No	Oncogene
F2_5	*INPPL1*	G1759T	No	Tumor suppressor gene
F2_5	*PIK3CA*	E545K	No	Oncogene
F2_5	*GNAI2*	G157T	No	ambiguous
F23_81	*TP53*	Y234C	Yes	Tumor suppressor gene
F27_97	*PIK3CA*	H1047R	Yes	Oncogene
F27_97	*BRCA1*	E597K	No	Tumor suppressor gene
F27_97	*TP53*	G244D	Yes	Tumor suppressor gene
F29_105	*STAT4*	C718T	No	ambiguous
F29_105	*GNA11*	L273F	No	Oncogene
F29_105	*TP53*	R248W	Yes	Tumor suppressor gene
F3_8	*CDKN1B*	F87S	No	Tumor suppressor gene
F3_8	*CD274*	G301C	No	Oncogene
F31_110	*BRCA2*	K2750Dfs*13	No	Tumor suppressor gene
F31_110	*TP53*	R249S	Yes	Tumor suppressor gene
F39_133	*CIITA*	G1660C	No	Oncogene
F39_133	*PTEN*	D92H	Yes	Tumor suppressor gene
F39_133	*TP53*	C238R	Yes	Tumor suppressor gene
F42_147	*TAGAP*	G1520C	No	ambiguous
F44_153	*TP53*	H168P	Yes	Tumor suppressor gene
F49_165	*BRCA1*	E597K	No	Tumor suppressor gene
F50_167	*BRCA2*	Q1089Sfs*10	No	Tumor suppressor gene

F50_167	*CIITA*	C1603A	No	Oncogene
F50_167	*MSH6*	R3L	No	Tumor suppressor gene
F50_167	*PDCD1*	G349T	No	ambiguous
F50_167	*XPC*	D586Y	No	Tumor suppressor gene
F50_167	*TP53*	Y220C	Yes	Tumor suppressor gene
F63_205	*TP53*	Splicing	No	Tumor suppressor gene
F65_208	*ACTB*	C899T	No	Oncogene
F65_208	*ATF2*	C850T	No	ambiguous
F65_208	*TP53*	Y163C	Yes	Tumor suppressor gene
F67_215	*TLR6*	N235delN	No	ambiguous
F85_261	*TP53*	C242Afs*5	No	Tumor suppressor gene
F9_27	*CDKN1B*	G25T	No	Tumor suppressor gene
F9_27	*NOTCH1*	S2516F	No	ambiguous
F9_27	*FANCD2*	P1224H	No	Tumor suppressor gene
F9_27	*FN1*	A621T	No	Tumor suppressor gene
F9_27	*PIK3CD*	G319T	No	Oncogene
F91_276	*ERCC6*	W1486C	No	ambiguous
F91_276	*PLCG1*	C262T	No	Oncogene
F92_281	*PIK3R1*	I82Sfs*32	No	Tumor suppressor gene
F92_281	*BRCA1*	C61Y	No	Tumor suppressor gene
F92_281	*TAF1*	T752C	No	Oncogene
F97_299	*PTEN*	R233*	No	Tumor suppressor gene
F97_299	*TP53*	A159V	Yes	Tumor suppressor gene

Supplementary table 3. Potential targeted treatments.

ID	Sample alteration	Drug	Gene tested/tumor type	Evidence	Source
F27_97	BRCA1 MUT (E597K)	PARP inhibitors (Olaparib,etc)	BRCA	Early trials	PMID:20609467;PMID:25366685
F27_97	BRCA1 MUT (E597K)	Platinum Agent (Chemotherapy)	BRCA	Early trials	PMID:25847936
F27_97	BRCA1 MUT (E597K)	Veliparib + Cisplatin (PARP inhibitor + Chemotherapy)	BRCA	Early trials	PMID:26801247
F27_97	PIK3CA MUT (H1047R)	AKT inhibitors	BRCA	Early trials	ASCO 2015 (abstr 2500)
F27_97	PIK3CA MUT (H1047R)	PIK3CA inhibitors	BRCA	Early trials	PMID:28331003
F2_5	HRAS MUT (G12D)	Tipifarnib (Farnesyltransferase inhibitor)	Any cancer type	Early trials	NCT02383927
F2_5	HRAS MUT (G12D)	Tipifarnib (Farnesyltransferase inhibitor)	Any cancer type	Early trials	NCT02383927
F2_5	PIK3CA MUT (E545K)	AKT inhibitors	BRCA	Early trials	ASCO 2015 (abstr 2500)
F2_5	PIK3CA MUT (E545K)	PIK3CA inhibitors	BRCA	Early trials	PMID:28331003
F3_8	CDKN1B MUT (F87S)	CDK2/4 inhibitors	Any cancer type	Pre-clinical Early trials	PMID:22471707 PMID: 30543440
F50_167	BRCA2 MUT (Q1089Sfs*10)	PARP inhibitors (Olaparib,etc)	BRCA	Early trials	PMID:20609467
F50_167	BRCA2 MUT (Q1089Sfs*10)	Platinum Agent (Chemotherapy)	BRCA	Early trials	PMID:25847936
F50_167	BRCA2 MUT (Q1089Sfs*10)	Veliparib + Cisplatin (PARP inhibitor + Chemotherapy)	BRCA	Early trials	PMID:26801247
F31_110	BRCA2 MUT (K2750Dfs*13)	PARP inhibitors (Olaparib,etc)	BRCA	Early trials	PMID:20609467
F31_110	BRCA2 MUT (K2750Dfs*13)	Platinum Agent (Chemotherapy)	BRCA	Early trials	PMID:25847936
F31_110	BRCA2 MUT (K2750Dfs*13)	Veliparib + Cisplatin (PARP inhibitor + Chemotherapy)	BRCA	Early trials	PMID:26801247

F101_313	BRCA1 MUT (G1077Afs*8)	PARP inhibitors (Olaparib,etc)	BRCA	Early trials	PMID:20609467;PMID:25366685
F101_313	BRCA1 MUT (G1077Afs*8)	Platinum Agent (Chemotherapy)	BRCA	Early trials	PMID:25847936
F101_313	BRCA1 MUT (G1077Afs*8)	Veliparib + Cisplatin (PARP inhibitor + Chemotherapy)	BRCA	Early trials	PMID:26801247
F92_281	BRCA1 MUT (C61Y)	PARP inhibitors (Olaparib,etc)	BRCA	Early trials	PMID:20609467;PMID:25366685
F92_281	BRCA1 MUT (C61Y)	Platinum Agent (Chemotherapy)	BRCA	Early trials	PMID:25847936
F92_281	BRCA1 MUT (C61Y)	Veliparib + Cisplatin (PARP inhibitor + Chemotherapy)	BRCA	Early trials	PMID:26801247
F9_27	CDKN1B MUT (G9W)	CDK2/4 inhibitors	Any cancer type	Pre-clinical	PMID:22471707
F49_165	BRCA1 MUT (E597K)	PARP inhibitors (Olaparib,etc)	BRCA	Early trials	PMID:20609467;PMID:25366685
F49_165	BRCA1 MUT (E597K)	Platinum Agent (Chemotherapy)	BRCA	Early trials	PMID:25847936
F49_165	BRCA1 MUT (E597K)	Veliparib + Cisplatin (PARP inhibitor + Chemotherapy)	BRCA	Early trials	PMID:26801247
F97_299	PTEN MUT (R233*)	ATM inhibitors	BRCA	Pre-clinical	PMID:27397505
F97_299	PTEN MUT (R233*)	Sirolimus (MTOR inhibitor)	Any cancer type	Early trials	ASCO 2013 (abstr 2532)
F39_133	PTEN MUT (D92H)	ATM inhibitors	BRCA	Pre-clinical	PMID:27397505
F39_133	PTEN MUT (D92H)	Sirolimus (MTOR inhibitor)	Any cancer type	Early trials	ASCO 2013 (abstr 2532)

15

Supplementary figures

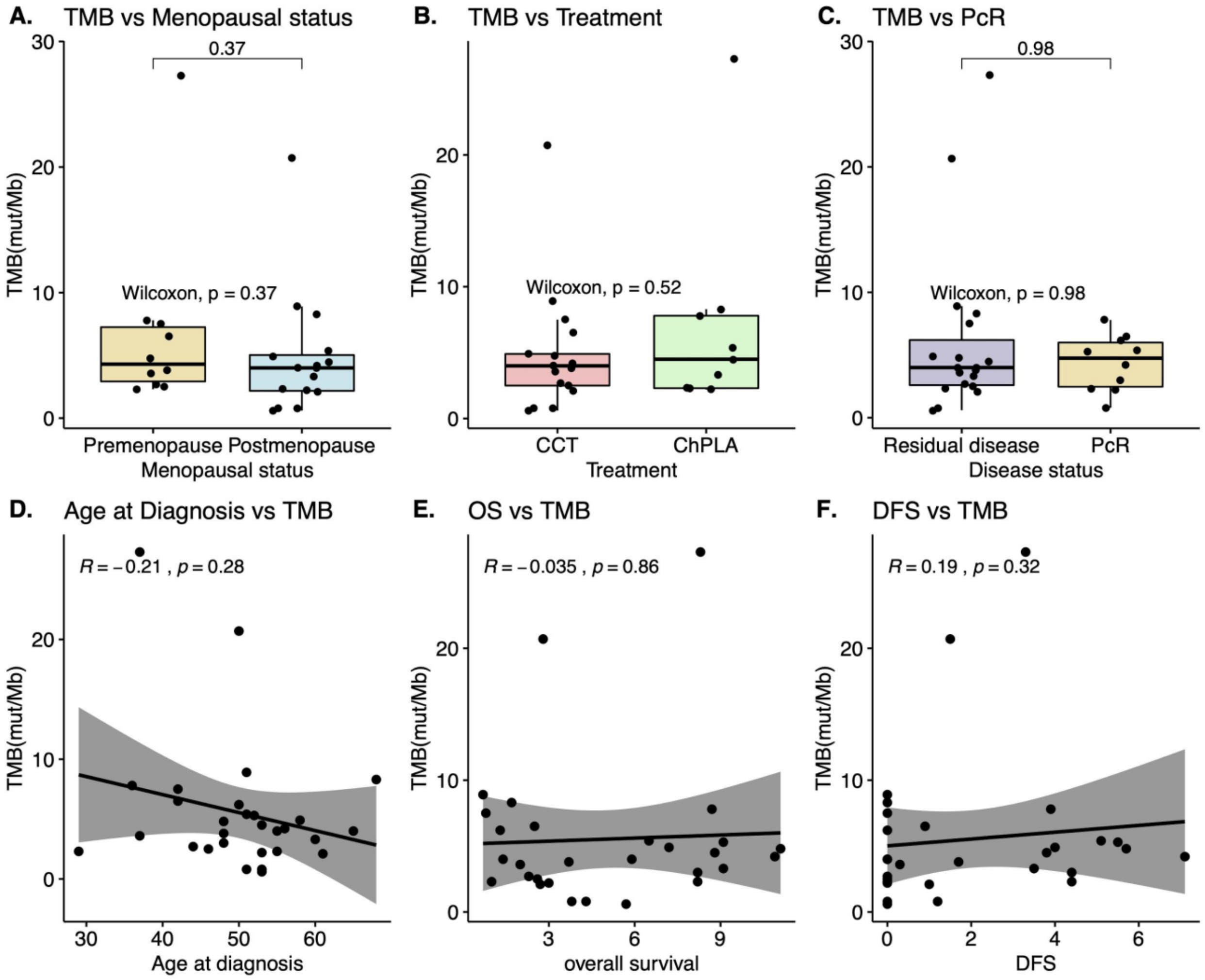

Supplementary figure 1. Association between clinical outcome and TMB. All the figures show the associations between the TMB and the clinical variables analyzed beginning with A. menopausal status. B. type of treatment C. PcR, D. Age at diagnosis, E. Os and F. DFS.

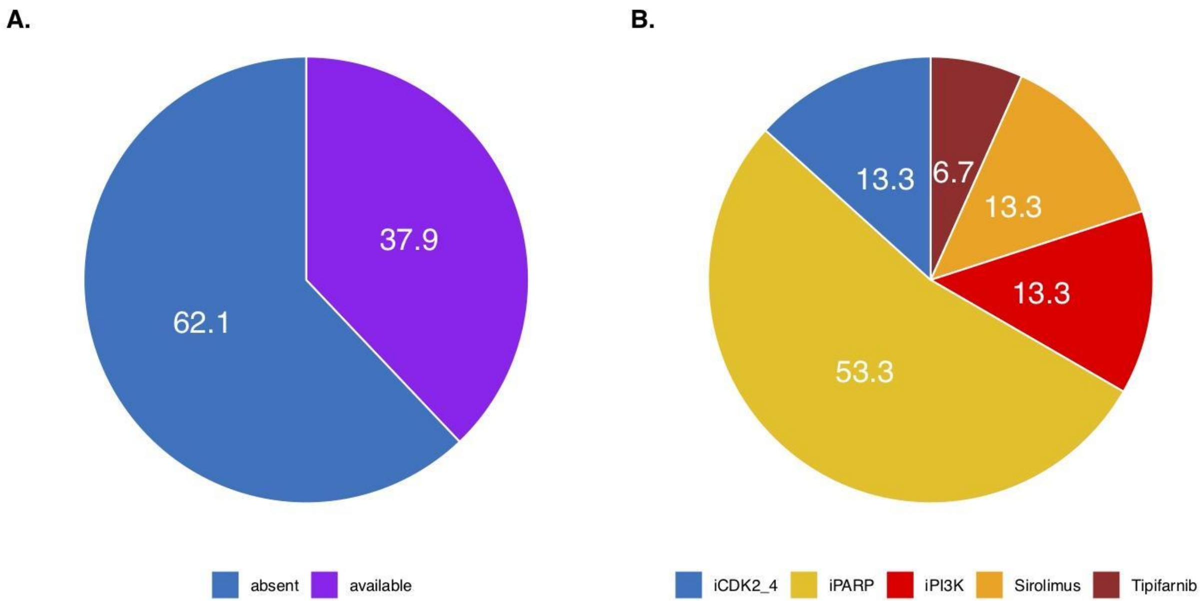

Supplementary figure 2. Targeted treatments A. percentage of patients with available targeted treatments and B. Percentage of the type of treatments available.

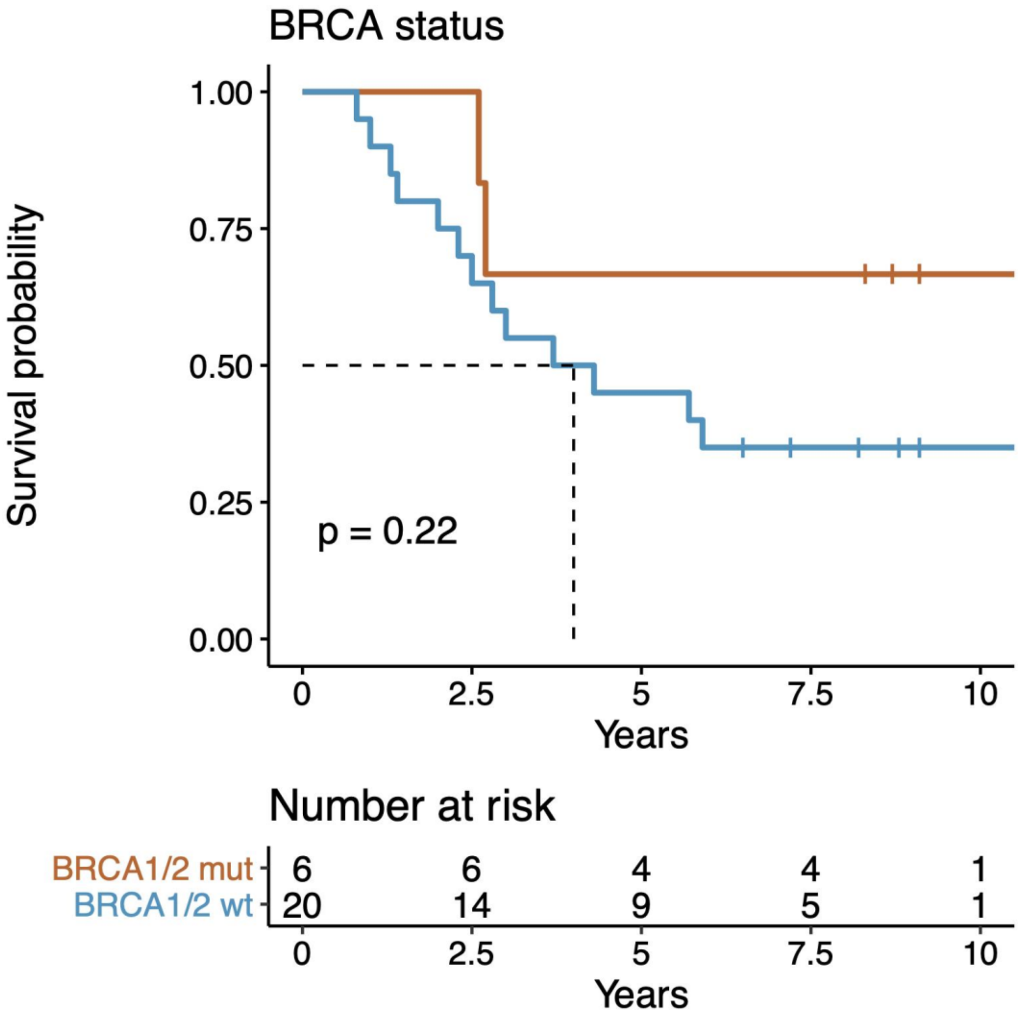

Supplementary figure 3. Overall survival as a function of *BRCA1/2* status. BRCA mutation showed a trend of higher OS.

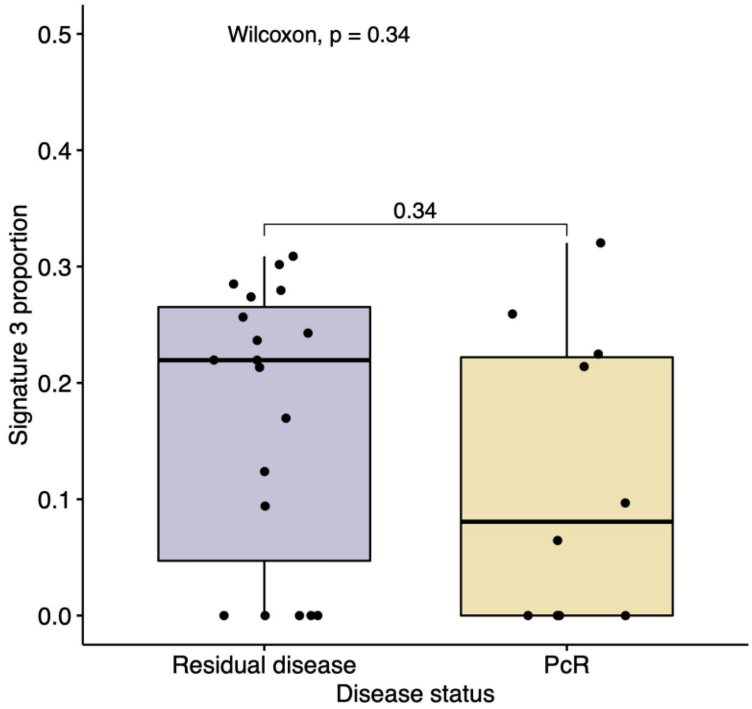

Supplementary figure 4. Association between PcR and signature 3.

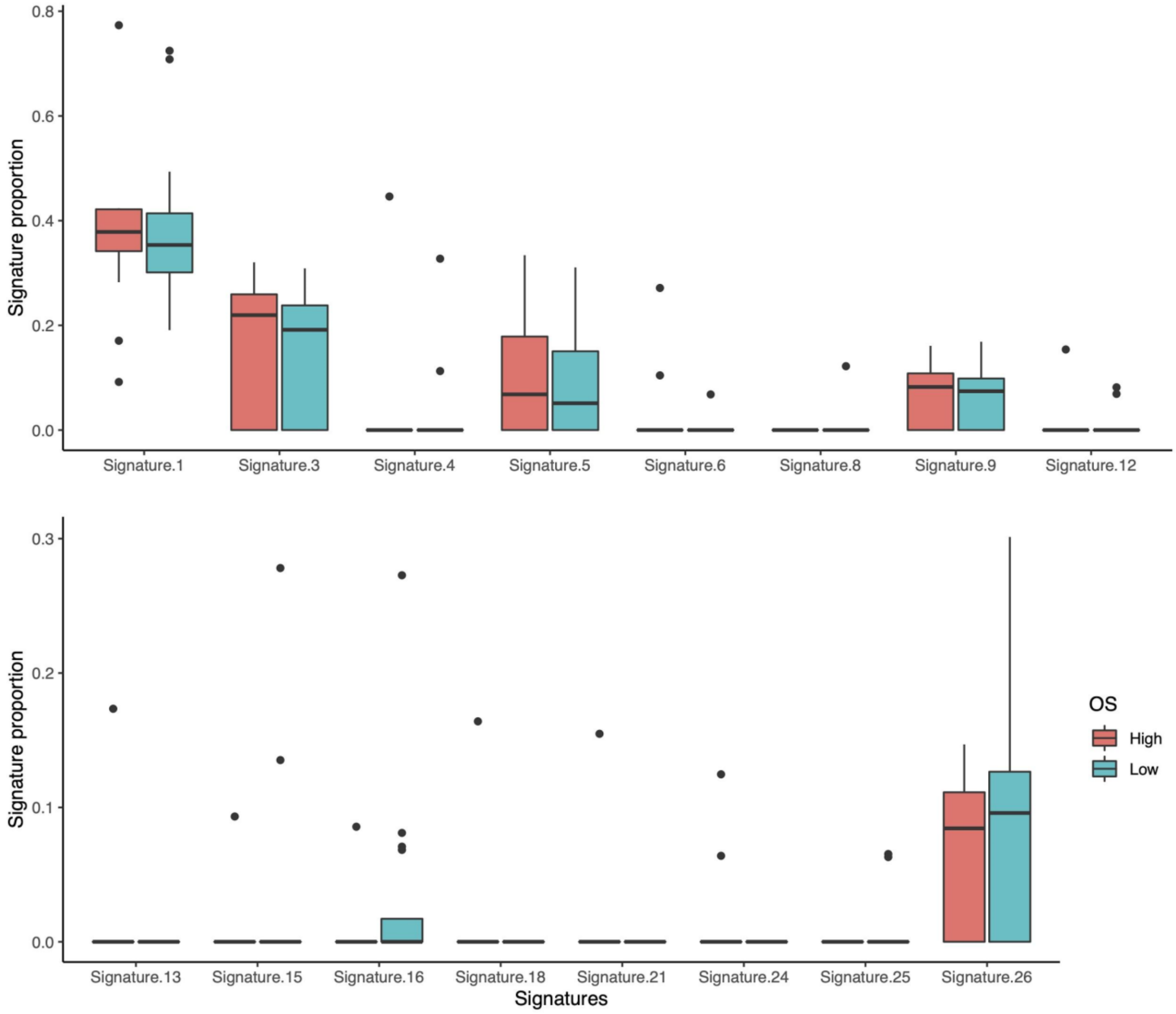

Supplementary figure 5. Comparison between the OS status (high and low) according to the signature proportion.

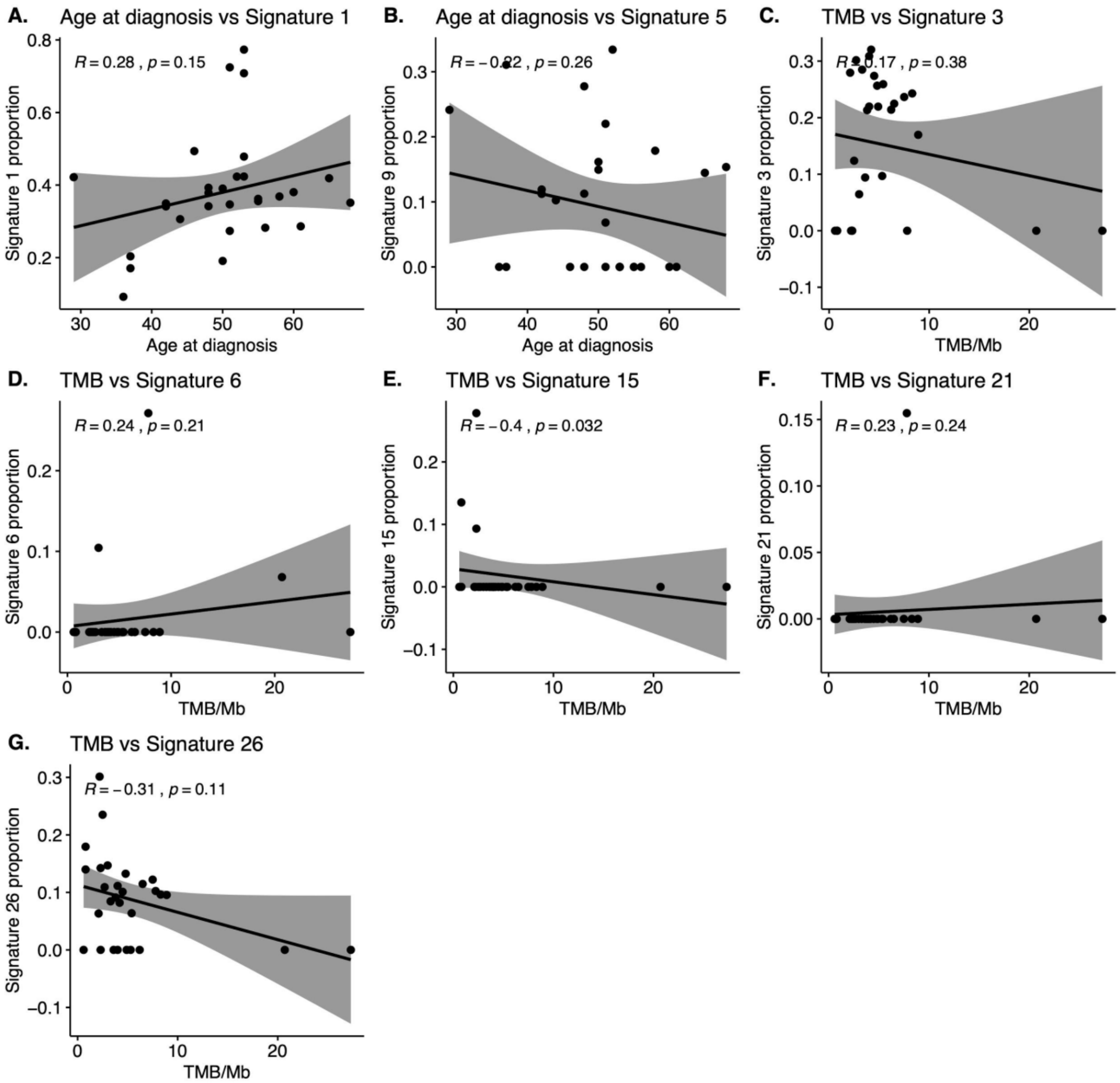

Supplementary figure 6. Linear correlation between signatures proportions with higher frequency in samples and clinical and molecular variables. Age at diagnosis vs A. signature 1 proportion and B. Signature 9 proportion. TMB vs C. signature 3, D. signature 6, E. signature 15, F. signature 21 and G. signature 26 proportion.

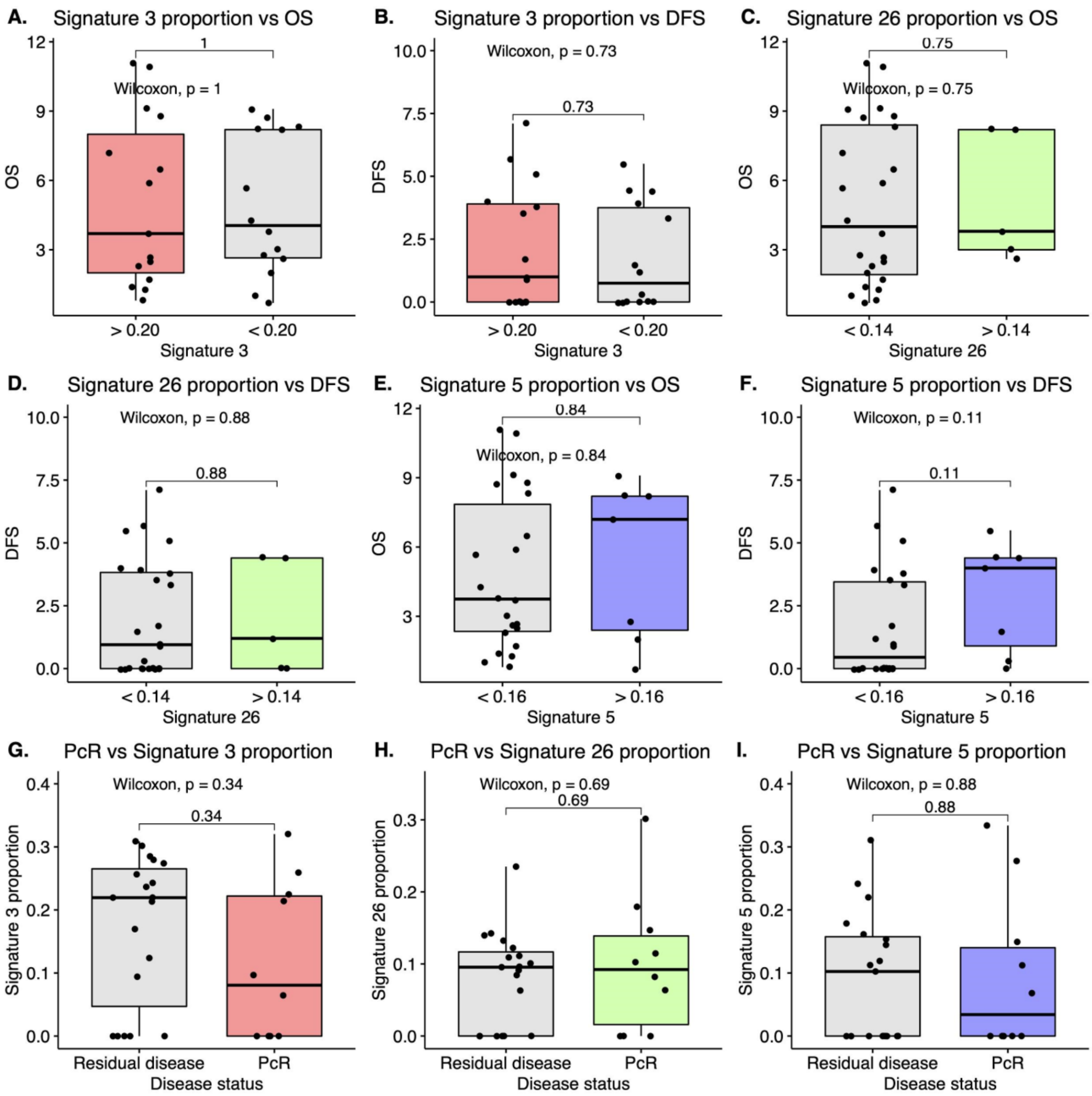

Supplementary figure 7. Association between threshold proportion in signatures according to the groups and the clinical outcome. OS and A. signature 3, C. signature 5 and E. signature 6. DFS and B. signature 3, D. signature 5 and F. signature 26. Red box indicated group A, green group B and blue group C; the gray boxes depicts the other two groups. The cutoffs of the proportions for each signature was selected by the threshold that divides each group.

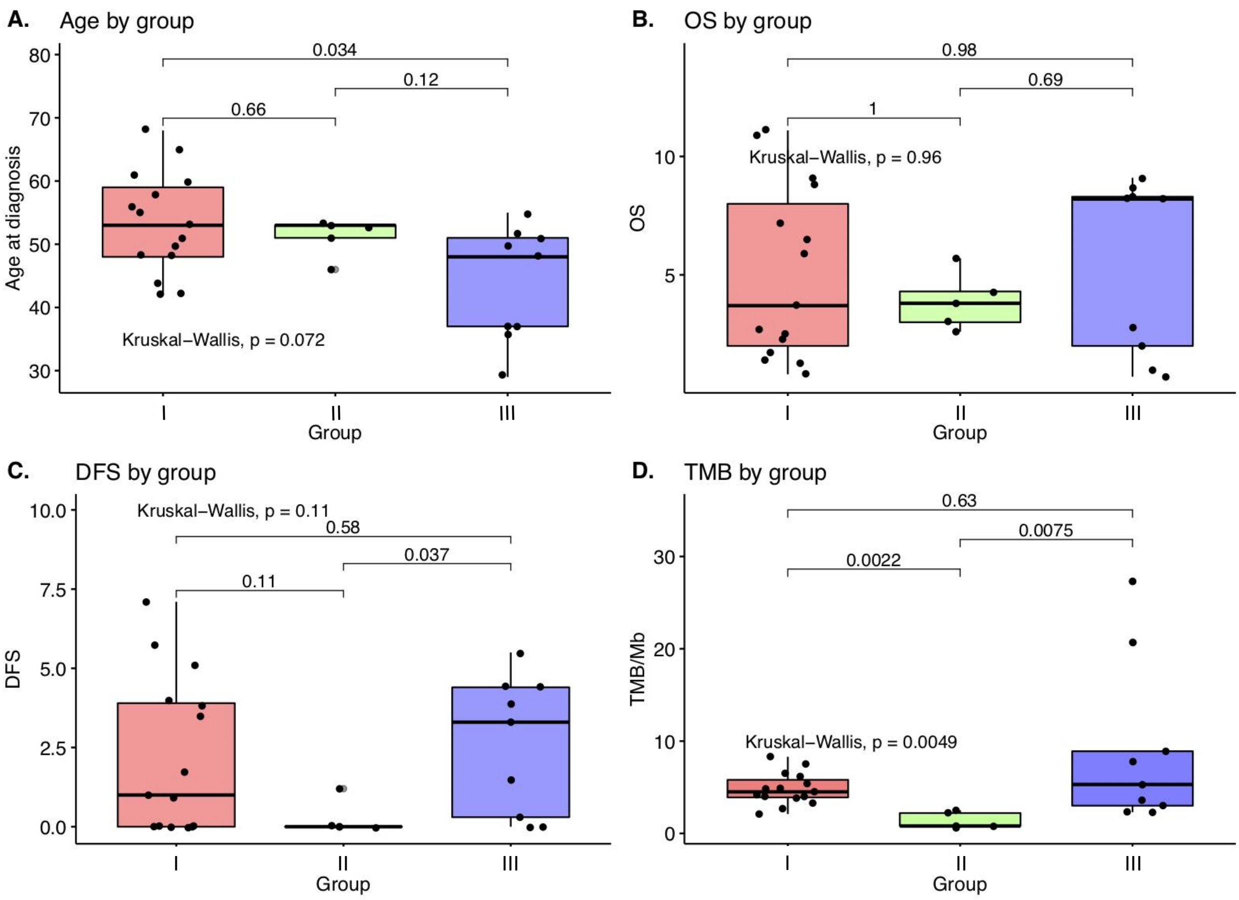

Supplementary figure 8. Differences between groups according to clinical (A, age at diagnosis; B, OS; C, DFS) and molecular outcome (D, TMB).

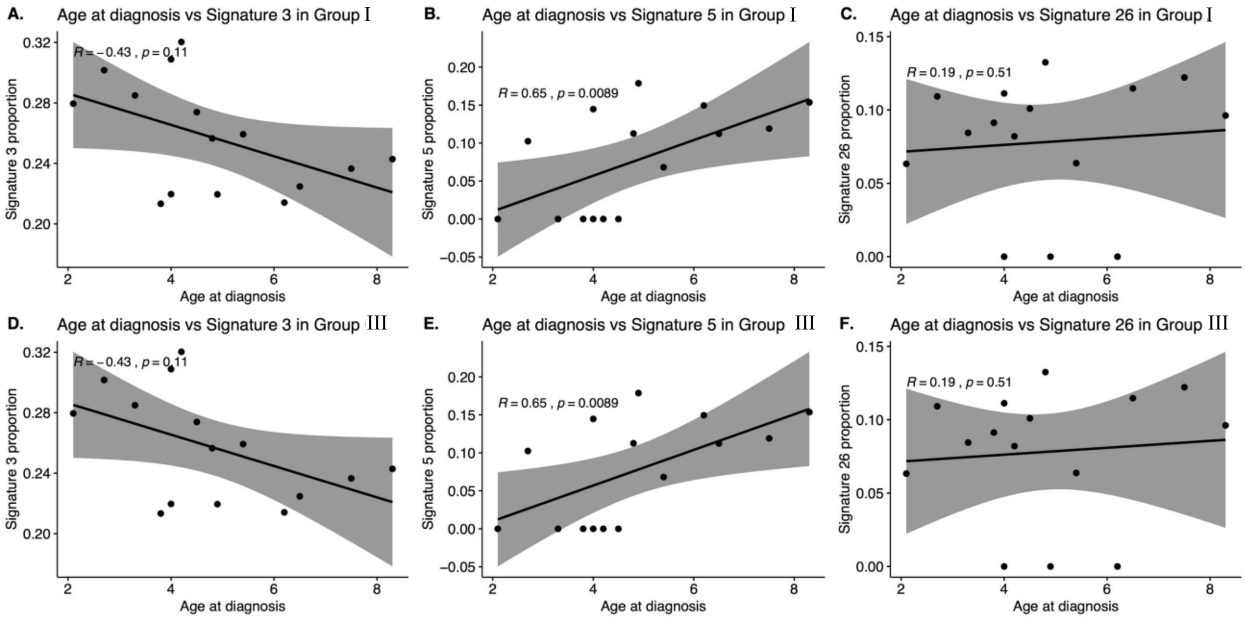

Supplementary figure 9. Linear correlation between age at diagnosis and signatures 3, 5, and 26 proportion according to signature groups I (A - C) and III (D - F).
